# Time Help Received During Crisis: Variations by Older Adults’ Cognitive Functioning

**DOI:** 10.1093/geroni/igaf122.1167

**Published:** 2025-12-31

**Authors:** I-Fen Lin, Judith Seltzer, Janecca Chin, Emily Wiemers, V Hotz

**Affiliations:** University of Missouri, Columbia, Missouri, United States; University of California, Los Angeles, Los Angeles, California, United States; University of Missouri, Columbia, Missouri, United States; Syracuse University, Syracuse, New York, United States; The University of Chicago, Chicago, Illinois, United States

## Abstract

Family caregiving is essential in meeting the care needs of older adults, particularly those with Alzheimer’s disease and related dementias (ADRD). The COVID-19 pandemic further disrupted an already fragile care landscape by decreasing the desirability of nursing home care, limiting the availability of paid caregivers, and destabilizing family caregiving arrangements. Older adults with ADRD are especially vulnerable to these disruptions, which can exacerbate their care needs and well-being. In this study, we used data from the 2020 Health and Retirement Study (HRS) to compare caregiving networks for older adults with and without ADRD, examining variations by age, sex, race-ethnicity, education, and partnership status. We analyzed verbatim responses from the COVID-19 module regarding time help received, creating new variables to capture helpers’ relationships beyond children and household members. Preliminary findings suggest that older adults with ADRD were more likely to include adult children in their caregiving networks, with notable demographic differences. Specifically, adults still in prime working ages (ages 55-64), men, Hispanics, and college-educated individuals with ADRD were more likely to rely on non-child helpers compared to their respective counterparts. This study advances prior research by expanding beyond the traditional focus on assistance with activities of daily living (ADLs) and instrumental activities of daily living (IADLs), as about one third of respondents with dementia in the HRS reported no ADL/IADL difficulties. Understanding the composition of caregiving networks for individuals with ADRD during a crisis is vital because these caregivers are likely to be called upon again when care needs increase.

